# Identification of a Novel c.3080delC JAG1 Gene Mutation Associated With Alagille Syndrome: Whole Exome Sequencing

**DOI:** 10.2196/33946

**Published:** 2022-07-08

**Authors:** Deepak Panwar, Vandana Lal, Atul Thatai

**Affiliations:** 1 Molecular Diagnostic Division National Reference Laboratory Dr Lal Pathlabs New Delhi India; 2 Molecular and Cytogenomics Division Max Specialty Hospital New Delhi India

**Keywords:** Alagille syndrome, JAG1, stop codon mutation, whole exome sequencing, gene database, clinical database, genome, genetics, sequence technology, diagnostic tool, genetic diagnosis, gene sequencing

## Abstract

**Background:**

Alagille syndrome is an autosomal dominant disorder associated with variable clinical phenotypic features including cholestasis, congenital heart defects, vertebral defects, and dysmorphic facies.

**Objective:**

Whole exome sequencing (WES) has become technically feasible due to the recent advances in next-generation sequencing technologies, therefore offering new possibilities for mutations or genes identification.

**Methods:**

WES was used to identify pathogenic variants, which may have significant prognostic implications for patients’ clinical presentation of the proband. In this paper, we have uncovered a novel *JAGGED1* gene (*JAG1*) mutation associated with Alagille syndrome in a 5-year-old girl presented with conjugated hyperbilirubinemia and infantile cholestasis.

**Results:**

The exome sequencing analysis revealed the presence of a novel *JAG1* heterozygous c.3080delC variant in exon 25. The detected variant introduced a stop codon (p.P1027RfsTer9) in the gene sequence, encoding a truncated protein. Our exome observations were confirmed through Sanger sequencing as well.

**Conclusions:**

Here, we report a case of a patient diagnosed with Alagille syndrome, conjugated hyperbilirubinemia, and infantile cholestasis, with emphasis on its association with the detection of the novel *JAG1* mutation, thereby establishing the genetic diagnosis of the disease.

## Introduction

Alagille syndrome (ALGS) is an autosomal dominant and multisystemic congenital disorder causing pediatric chronic liver disease with the prevalence of 1: 70,000-100,000 in infants [1,2]. Alagille syndrome (ALGS; MIM: 118450) is characterized by intrahepatic bile ducts, highly variable clinical features, including cholestasis, skeletal malformations, cardiac, ocular abnormalities, and dysmorphic facial features [1]. The classical diagnosis of ALGS includes low numbers of hepatic bile ducts, which results in chronic cholestasis leading to cirrhosis and end-stage liver disease. Hepatic manifestations are variable, and some patients present with jaundice, though it does not progress to a more serious disease [3].

ALGS is caused by mutations in either the *JAGGED1* (*JAG1*) or *NOTCH2* gene*.* Both these genes are involved in the Notch signaling pathway and play an important role in transcription regulation and cell fate determination [4-7]. The majority of ALGS cases (∼97%) are caused by mutations in *JAG1* (MIM: 601920) gene, while less than 1% of patients have a heterozygous mutation in the *NOTCH2* gene (1p13) [8,9]. The *JAG1* gene is located on chromosome 20 (20p12.2), encompassing 26 exons that encode a 1218 amino acid protein that participates in the Notch signaling pathway as a ligand [10]. Phenotypic effects of JAG1 mutation in ALGS are highly variable with reduced penetrance. However approximately 94% of patients with a clinically confirmed diagnosis of ALGS carry JAG1 mutations [11]. There is a high rate of de novo mutations*,* with approximately 60%-70% of mutations in probands not found in either parent [12-15].

It has been reported that the pathogenic mutations of in *JAG1* include missense mutations (11%), nonsense and frame-shift mutations (69%), splice site mutations (16%), and deletion of the entire *JAG1* gene (4%) [12,14,16-23]. *JAG1* mutations in ALGS clinical presentation have been reported in various populations, such as American, European, Australian, and Japanese [12,14,16-23], whereas there are only few clinical studies on ALGS from India [24-26]. Because of the wide range of clinical manifestations, early genetic testing is required to establish the condition and to take preventative steps to avoid consequences in numerous organs. The advent of molecular diagnostic testing has led to a revision of diagnostic criteria for ALGS [1]. Next-generation sequencing analysis, including either genome or exome sequences, have been recommended for the molecular diagnosis of neonatal or infantile intrahepatic cholestasis [22]. Whole exome sequencing (WES) allows sequencing of all expressed genes in the genome, which is substantial, considering the protein-coding regions cover approximately 85% of human disease-causing mutations [27]. In this study, using WES, we identified a novel *JAG1* mutation associated with early onset of Alagille syndrome.

## Methods

### Case Presentation

The proband is a 5-year-old girl presented with conjugated hyperbilirubinemia and infantile cholestasis with the onset of clinical manifestation of features at 10 months of age.

### Ethics Approval

The study design and protocol were conducted in accordance with the guidelines of the American College of Medical Genetics and Genomics and was approved by the Ethical Review Committee of Dr Lal Pathlabs. Written informed consent has been taken from parents of the proband included in the study, and the parents have provided consent to publish the data.

### Library Preparation and WES

The DNA was extracted from 2 ml of the peripheral blood using Qiagen DNA mini kit, as per the manufacturer’s instructions. The quantity and quality of the extracted genomic DNA were measured by NanoDrop-2000 Spectrophotometer. Approximately 100 ng of genomic DNA was used to construct exome library using Ion Ampliseq Exome RDY Panel kit (Thermo Fisher Scientific). The resulting DNA library was quantified with Qubit dsDNA HS (High Sensitivity) Assay Kit on Qubit 3.0 Fluorometer. Approximately 25 pm of the library was used with the Ion Chef Instrument (Thermo Fisher Scientific) for template generation followed by enrichment of the templated ion sphere particles. Sequencing was performed using Hi-Q chemistry on Ion Proton system (Thermo Fisher Scientific).

### Data Processing and Variant Analysis

The sequences were aligned against the reference genome (GRCh37/hg19) in Torrent Suite v.5.12.0 and Torrent Suite Variant Caller v.5.2.1 software (Thermo Fisher Scientific) with default parameters. The coverage analysis plugin and variant caller plugin from Life Technologies (Thermo Fisher) were used to analyze the Ion Proton sequencing run. Variant discovery, genotype calling of multiallelic substitutions, and indels were performed on each individual sample using the Torrent Variant Caller (TVC, version 4.6.0.7; Thermo Fisher). Statistics and graphs describing the level of sequence coverage produced for targeted genomic regions were provided by the Torrent Coverage Analysis (version 4.6.0.3). The variants were annotated by the Annotate variants 5.0 of Ion Reporter (Thermo Fisher).

### Variant Prioritization and Bioinformatics Analysis

Variants that were detected in the exome sequencing were filtered based on coverage (≥15x), minor allele frequency (≤0.01), and deleterious potential. All resulting variants were contrasted with the Human Gene Mutation Database [28] and Uniprot [29]. Furthermore, intronic, up- or downstream, and synonymous variants were removed. The pathogenicity of the detected variant was evaluated using Mutation Taster [30] and MutPredLOF [31]. Additional factors that were considered include the following: (1) absence in the general population; (2) novel appearance and disease phenotype from the family pedigree; (3) absence of any other mutation in *JAG1* that could be responsible for the clinical phenotype; and (4) previous independent occurrence in an unrelated patient. An Integrative Genome Viewer [32] was used to visualize sequencing data. Variant frequencies were obtained from various databases such as the 1000 Genomes Project, dbSNP142, Exome Aggregation Consortium (ExAC) and gnomAD. Finally, for the interpretation of variant, American College of Medical Genetics and Genomics 2015 guidelines were used [33].

### Mutation Confirmation: Sanger Sequencing

Confirmation of the mutation was performed by conventional Sanger sequencing using the BigDye Terminator v3.1 Cycle Sequencing kit (Applied Biosystems, Thermo Fisher Scientific) and was loaded on an ABI 3500Dx automated Genetic Analyzer (Applied Biosystems, Thermo Fisher Scientific). Primer sequences for the identified variant were designed using Primer 3.0 online as follows: Frd 5′- CCTCATTATTCGATGGCAAGGC -3′ and Rev 5′- GTTCTGTTCTTCAGAGGCCG-3′.

## Results

### Whole Exome Sequencing Analysis

**Figure 1:**
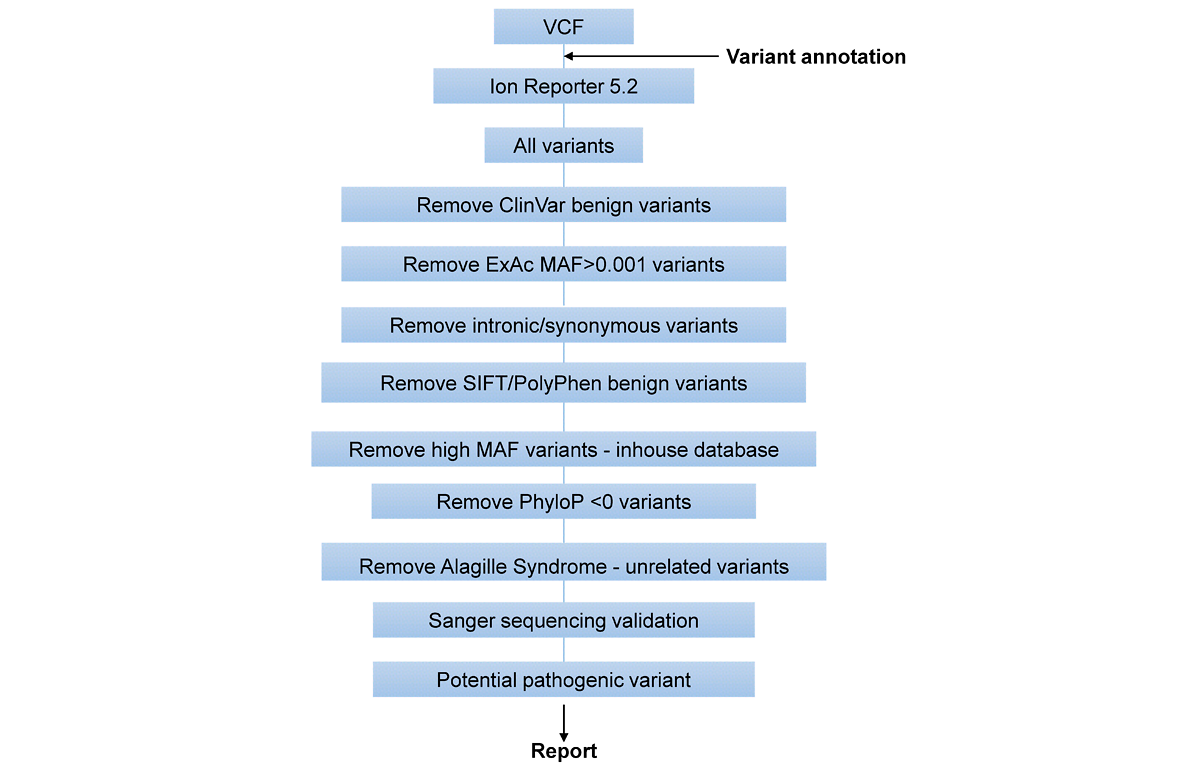
Illustration for the variant filtering process in whole exome sequencing . ExAC: Exome Aggregation Consortium; MAF: mutation annotation format. VCF: variant call format.

We detected a total of 39,679 variants comprising 55% (n=21,823) synonymous, 43% (n=17,062) missense, and 2% (n=794) frameshift or indel variants. For WES data filtering procedures, a filtering tree illustrated the step-by-step narrowing down of candidate gene or variants detected during next-generation sequencing data analysis (Figure 1). The first phase consisted of benign and synonymous variant filtering, and the second phase was based on variant impact (nonsynonymous and truncating), allele frequency (<0.1%), and pathogenicity prediction tools for missense variants (score >3). Since there were still a high number of candidate variants and genes, a second round of prioritization based on manual curation of biological function was performed, and variants in genes unrelated to ALGS were filtered out.

**Figure 2:**
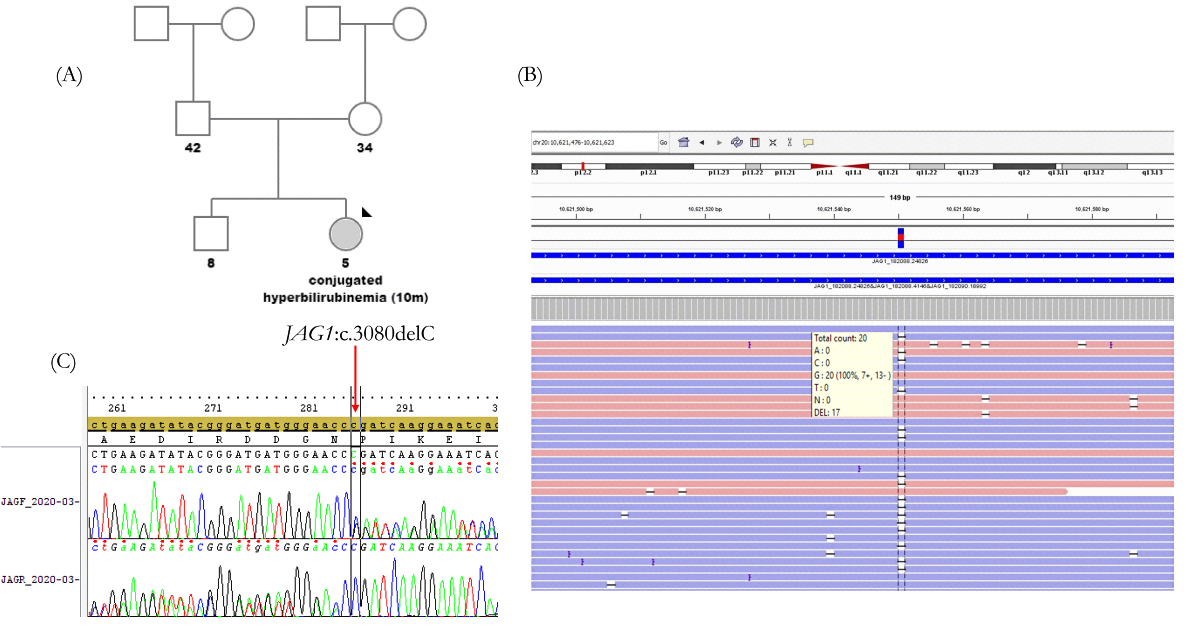
Proband’s pedigree and electropherogram of identified disease associated variant. (A) Family pedigree of patient diagnosed with Alagille Syndrome. (B) IGV plot showing the mutation region in WES data in the proband. Track comprises two parts: a histogram of the read depth and the reads as aligned to the reference sequence. Reads are colored according to the aligned strand (red=forward strand; blue=reverse strand). (C) Sanger sequencing confirmation of heterozygous *JAG1* variant c.3080delC in the patient. A: adenine; C: cytosine; DEL: deletion; G: guanine; JAGF: *JAG1* forward primer; JAGR: *JAG1* reverse primer; T: thymine.

WES results indicated a novel heterozygous frameshift variant (c.3080delC;p.P1027RfsTer9) in the *JAG1* gene responsible for ALGS (Figure 2A). The sequence alignment of heterozygous deletion (c.3080delC) at position Chr20:10621549 in *JAG1* gene was viewed using the Integrative Genomics Viewer (Figure 2B). Sanger sequencing analysis confirmed that the proband carried the mutation in a heterozygous state (Figure 2C). This mutation is conserved across different species and can greatly affect the amino acid sequence of *JAG1* gene that might change the protein function. Different web-based bioinformatics tools were used to analyze the pathogenicity of the variant and predicted this mutation to have potential damaging effects (Table 1).

**Table 1:** Whole exome sequencing analysis identified the *JAGGED1* gene (*JAG1*) mutation in the proband.

Locus	Gene	Exon	Protein	Coding	Mutation Taster^a^	MutPredLOF^a^
Chr20:10621549	*JAG1*	25	p.Pro1027fs	c.3080delC	D^b^	D

^a^Mutation Taster and MutPredLOF are functional prediction scores in which increasing values indicate a more damaging effect.

^b^D: damaging or deleterious.

## Discussion

### Principal Findings

ALGS is a highly variable autosomal dominant disorder, which involves multiple organ systems, and it requires a multidisciplinary team of medical specialists for its management [33-35]. The spectrum of mutations in *JAG1* gene associated with ALGS includes full gene deletion and other protein-truncating mutations including nonsense, frameshift, and splice site as well as missense mutations, suggesting that the clinical phenotype is caused by haploinsufficiency for the *JAG1* protein [9,15]. Phenotypic effects of *JAG1* mutations are highly penetrant but with variable expressivity [36]. There is no strong correlation between the type and location of the *JAG1* mutation and the severity of the disease, suggesting that other genomic modifiers beyond the known *JAG1* mutation may be the cause of the variable expressivity that characterizes this disorder [9]. Unfortunately, no genotype-phenotype correlation exists between clinical manifestations and the specific *JAG1* pathogenic variant or the location of the mutation within gene [37]. Although genetics of ALGS is well-defined, there is variable expressivity of the disease. Individuals with the same mutations, including patients belonging to the same family, show discordance in the phenotype [38]. In support of this concept, the genotype-phenotype correlation studies did not identify a link between the mutation type and clinical manifestation or severity [39].

**Figure 3:**
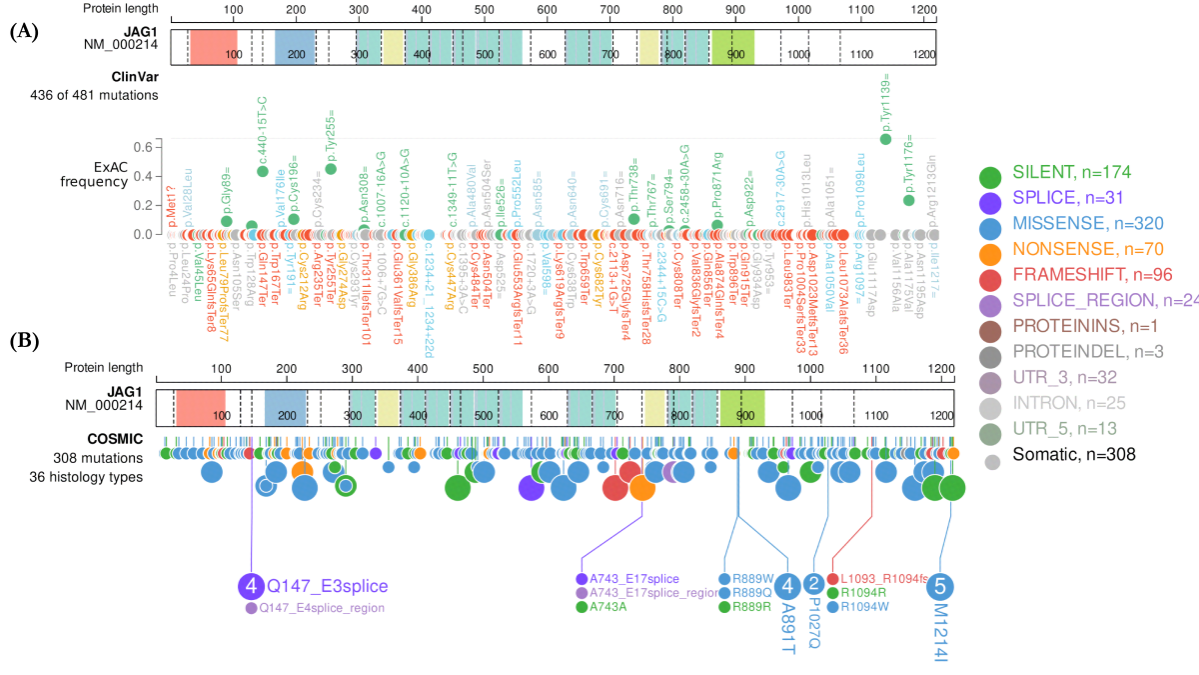
Schematic of JAG1 protein with all listed variants: (A) ClinVar and (B) COSMIC databases. Dashed lines within the protein indicate exon boundaries, and numbers indicate amino acid coordinates. Protein domains include (*JAG1*): N terminus signal peptide (Salmon), DSL domain (Skyblue), EGF‐like domain (teal), EGF domain (yellow), and VWC out domain (light green). All mutations listed are shown in different colors. *JAG1*: JAGGED 1 gene; ExAC: Exome Aggregation Consortium; DSL: Delta serrate ligand; EGF: EGF-like domain; UTR: Untranslated Region; VWC: von Willebrand factor (vWF) type C domain.

To the best of our knowledge, we report here a novel variant underlining a frameshift mutation in the *JAG1* gene using the Ion Torrent platform. In the patient currently under study, the onset of ALGS was at an early age with hyperbilirubinemia and infantile cholestasis. WES analysis revealed the previously unreported heterozygous c.3080delC variant in exon 25, which produces a truncated *JAGGED1* protein due to a stop codon (p.P1027RfsTer9) and probably a diminished function of the Notch signaling pathway. A study reported that some symptoms associated with ALGS are deemed indicators of a bad prognosis, such as high total bilirubin levels between 12 and 24 months of age, liver fibrosis, and xanthomata [40]. The present patient had one of these predictors, high total bilirubin. However, further studies could clarify the effect of this mutation on the protein and signaling level. This *JAG1* (c.3080delC) mutation has never been reported in public human databases, including the following: ClinVar [41], COSMIC [42], the 1000 Genomes Project [43], gnomAD [44], ExAC [45], dbSNP [46], and the Human Gene Mutation Database [28]. It was also not found in our in-house database of 1000 exomes (personal data). In Figure 3A and B, we illustrated all *JAG1* pathogenic, or likely pathogenic, mutations reported in the public version of ClinVar and COSMIC databases according to ExAC frequency. These variants encompass frameshift (nucleotide‐level deletions, insertions, and insertion-deletions), nonsense (substitutions, start loss, and stop gain), missense, splice site, and in-frame deletions. We observed that the effect of mutations on phenotype and its severity differs between patients regardless of their mode of inheritance, which makes the understanding of the physiopathological mechanisms so far unknown. Interestingly, in the same exon 25, a missense mutation c.3080C>A;p.Pro1027Gln was previously reported in the COSMIC database (Figure 3B) which was disease-causing according to MutationTaster and damaging according to other in silico tools; FATHMM [47], MutPred [31], LRT [48], and EIGEN PC [49]. A 2019 study by Gilbert et al [50] showed that 94.3% of individuals with clinically diagnosed ALGS have a pathogenic variant in the *JAG1* gene, 2.5% have a pathogenic variant in the NOTCH2 gene, and 3.2% are molecularly uncharacterized [50]. The spectrum of *JAG1* mutations includes more frequently protein-truncating mutations (75%) and nonprotein truncating mutations (25%) [38,51].

In summary, our report broadens the spectrum of mutations in the *JAG1* gene. It thus aids the geneticist for better identification of the etiological mutations leading to ALGS, thereby providing further insights to detect the associated hot spots, exons or mutations.

### Conclusions

WES has revolutionized molecular genetic research and has become an essential genetic tool for molecular diagnosis of heterogeneous disorders. This study is an attempt to improve our understanding of the origin of ALGS caused by the identification of a variant in the *JAG1* gene. The novel mutation identified here provides an appropriate course of management to the patient to offer genetic counseling to the family; it also offers to expand the genetic spectrum of *JAG1*-related ALGS, raise awareness among pediatricians on the morbidity of this severe form of ALGS, which is thus far underdiagnosed, and show them the added value of next-generation sequencing technology in reducing diagnostic wandering.

## Abbreviations

ALGS: Alagille syndrome
ExAC: Exome Aggregation Consortium
*JAG1*: *JAGGED1* gene
NOTCH2: notch receptor 2
WES: whole exome sequencing

## Data Availability Statement

The data used to support the findings of this study are included within the article.
